# Methods used to develop quality of care standards and indicators for mental health across the WHO European region: a rapid systematic review

**DOI:** 10.1136/bmjoq-2025-003533

**Published:** 2025-12-09

**Authors:** Jennifer Hall, Raffaella Sibilio, Ledia Lazeri, Joao Breda

**Affiliations:** 1 WHO Office on Quality of Care and Patient Safety, Athens, Greece; 2 WHO Regional Office for Europe, Copenhagen, Denmark

**Keywords:** Mental health, Quality measurement, Quality improvement methodologies

## Abstract

**Objective:**

This rapid review aims to understand whether a standardised approach to developing quality standards and indicators for mental health has been used across the WHO European Region and beyond to inform methods to develop quality standards for child and adolescent mental health services.

**Introduction:**

Improving the quality of child and adolescent mental healthcare across the WHO European Region is a priority. Despite advances in quality of care for mental health, many challenges remain, including the lack of a standardised approach to quality improvement.

**Inclusion criteria:**

Papers that outline methods used to develop quality standards or indicators for mental healthcare, published since the year 2000 in English and for use in the WHO European Region, Australia, Canada or the USA, were included.

**Methods:**

Methods were based on guidance from the Joanna Briggs Institute and WHO. Searches were conducted across PubMed, Scopus, PsycInfo and Google Scholar from 16 January to 30 January 2024. The titles/abstracts and full-text articles were screened by two reviewers independently, and the inclusion/exclusion criteria were applied. A template based on five steps proposed to develop health indicators was used to extract relevant data by one reviewer and verified by another.

**Results:**

21 studies were included in the review. All papers originated from high-income countries, with dominance from the USA, Canada and the UK. Most papers described four or five of the five proposed steps; however, there was variation in the extent to which these steps were described and how they were implemented.

**Discussion:**

The results suggest that no consistent approach has been used to develop quality standards/indicators for mental healthcare. There is a need for more participation from people with lived experience and for more research across a wider geographic area.

**PRSOPERO registration number:**

CRD42024496509.

**WHAT IS ALREADY KNOWN ON THIS TOPIC**

**WHAT THIS STUDY ADDS**

- There is a lack of consensus for developing mental healthcare quality standards and indicators. Differences were seen in how the methods themselves are created, how to define the conceptual framework, how to find potential quality standards and indicators, the criteria for selecting indicators and standards, consensus-building methods, whether a pilot was used and what stakeholders are involved in the process.

**HOW THIS STUDY MIGHT AFFECT RESEARCH, PRACTICE OR POLICY**

- There is a need to standardise the process to develop quality standards and indicators for mental health. Future methods should include the development of a steering group, the use of agreed criteria to prioritise standards/indicators (eg, feasibility and importance), the use of a pilot process and the involvement of people with lived experience throughout.

## Introduction

 In the WHO European Region, 14% of children and adolescents experience a mental disorder^1^ and suicide is the leading cause of death among those aged 15–29 years.^2^ Over time, self-reported well-being is decreasing^3^ and the number of children and adolescents living with a mental disorder is increasing.^1^ Despite this high and increasing need, most children and young people with mental health conditions do not access support^4^ and the quality of care is variable.^5^

Quality health services should be effective, safe, people-centred, timely, equitable, integrated and efficient.^6^ However, the quality of mental health services often lags behind that of physical health services.^7^ This disparity may be more pronounced for child and adolescent mental health services, which often receive less funding than adult services.^8^ Challenges to high-quality child and youth mental health (CAYMH) care include a lack of specific mental health policies and plans for this population,^8^ human rights abuses,^9^ lack of appropriate services^8^ and a shortage of adequately trained healthcare workers.^10^

In response, initiatives to improve the quality of mental healthcare have not progressed as far as those for general health services.^11^ For example, quality standards and indicators for CAYMH exist in only a few high-income countries globally,^12^,^14^ and not enough is known about how to effectively implement quality improvement for CAYMH care.^15^ Quality standards define high-quality care in measurable statements, and quality indicators provide a way to measure that statement.^12^,^14^

It is not surprising that there have been calls for more investment and research to improve the quality of CAYMH care.^7^ Stakeholders from the WHO European Region (‘the Region’) highlighted the need to improve the quality of CAYMH care and, particularly, for quality standards to allow for a unified approach to assess and measure the quality of CAYMH mental health services.^16^

As a result, the WHO Regional Office for Europe is developing quality standards for CAYMH services. The first step is to create the methods for their development. Initial searches yielded no guidance documents outlining methods to develop quality standards or indicators for mental health services; however, several frameworks to support the development of global health quality measures were found.^17 18^ However, these documents did not outline a consistent approach that could be easily adapted for our purposes.

Without an easy-to-adapt guidance document, it was decided to understand what methods have been used previously to develop mental healthcare quality indicators and standards. Initial searches yielded very few results for CAYMH care, hence the searches were broadened to include all age groups. These learnings can be used to inform subsequent methods to develop quality standards and indicators for mental healthcare and other healthcare more broadly in the future.

This rapid review aims to understand what methods have been used to develop quality standards and indicators for mental healthcare and whether a consistent approach has been used.

## Methods

A rapid review allows for an efficient overview of the literature within a constrained time frame. Here, it was used to develop a snapshot of the current research on the development of quality standards and indicators for mental healthcare in the Region, including both published and grey literature.

This rapid review has been conducted in accordance with the *Joanna Briggs Institute Manual for Evidence Synthesis*^19^ WHO guidance on *Rapid reviews to strengthen health policy and systems: a practical guide.*^20^ The protocol of this review has been registered on Prospero (ID: CRD42024496509).

No ethical approval was sought, as only publicly accessible documents were used as evidence. No direct contact was had with any human subjects or data. Patients or the public were not involved in the design, conduct, reporting or dissemination plans of our research.

### Search strategy

**Figure 1:**
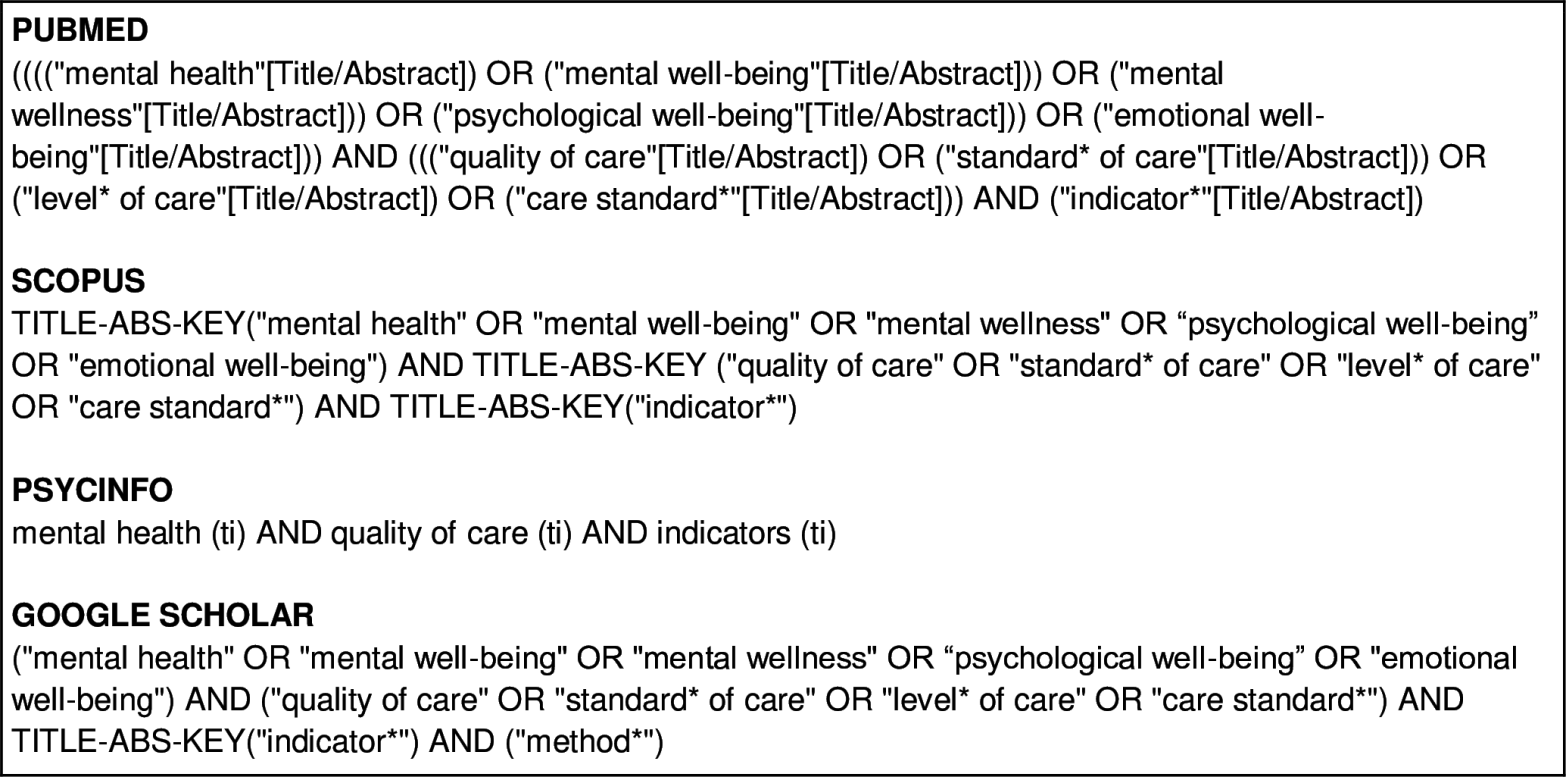
Full search strategy.

An initial limited search of PUBMED and SCOPUS was undertaken to identify articles that met the inclusion/exclusion criteria. Paper titles, abstracts and index terms were used to develop a full search strategy for use in PUBMED, SCOPUS, PSYCINFO and GOOGLE SCHOLAR (see figure 1 for the full search strategy). The search strategy was adapted as needed for each database and/or information source.

The systematic search was conducted across PubMed, Scopus, PsycInfo and Google Scholar from 16 January to 30 January 2024 by one researcher (JH), using three groups of key words related to the terms “mental health,” “measures” (indicators, standards) and “quality of care”. These three categories were combined using the Boolean “AND” and “OR.” The reference lists of all included papers were manually screened for additional studies that may meet the inclusion/exclusion criteria. An additional search for grey literature was conducted on Ministry of Health websites and Google search engine.

**Figure 2:**
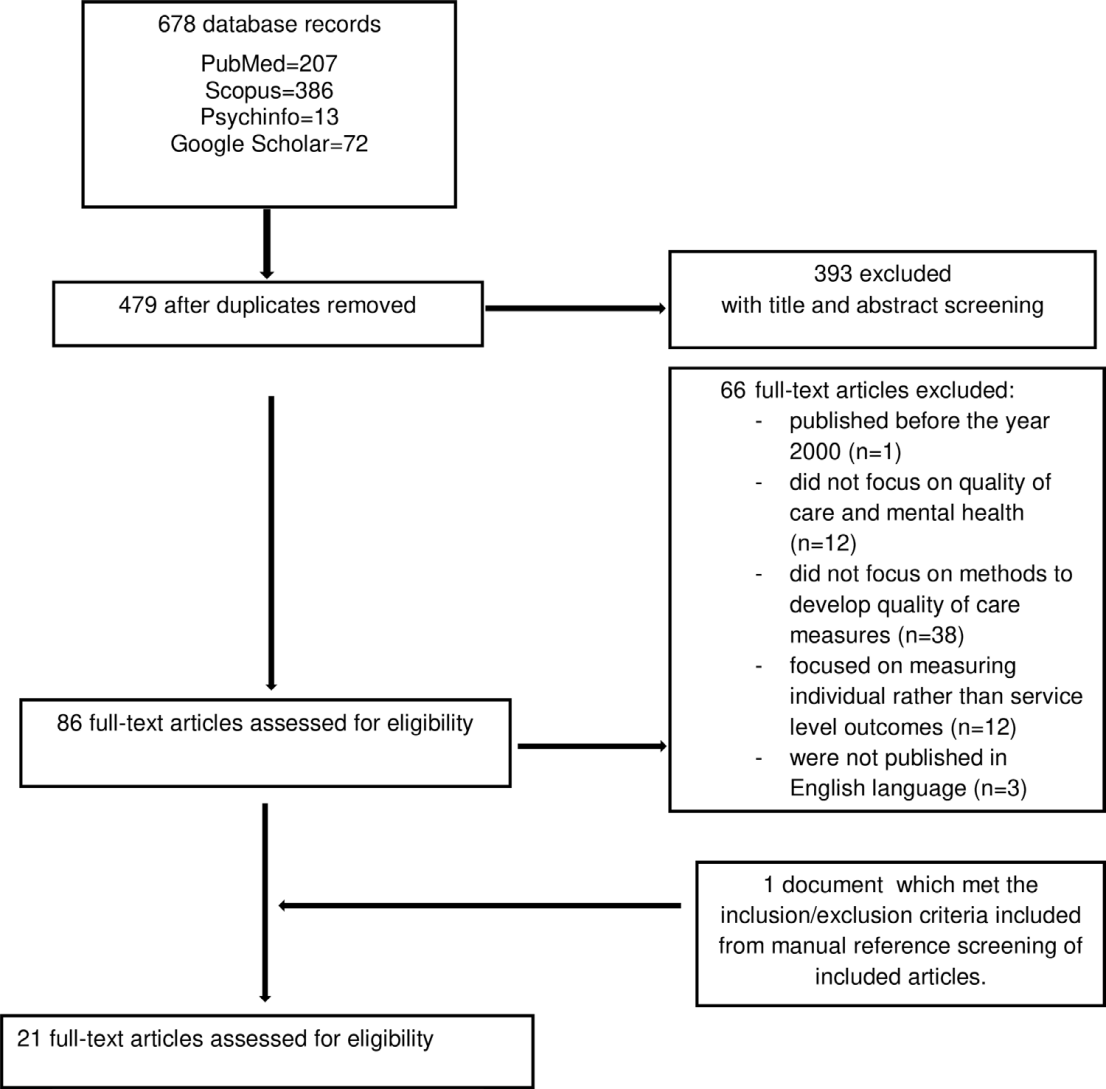
Flowchart of articles.

All identified papers were collated, and duplicates were removed. Titles and abstracts of the articles were screened independently by two reviewers (JH and RS) for assessment against the inclusion/exclusion criteria. The remaining articles were subject to a full-text review against the inclusion/exclusion criteria, and reasons for exclusion were recorded. Any disagreements that arose between the reviewers (JH and RS) at each stage of the selection process were resolved through consensus-building discussions with the option for third reviewer arbitration. The results of the search and the study inclusion process are reported in figure 2.

**Figure 3:**
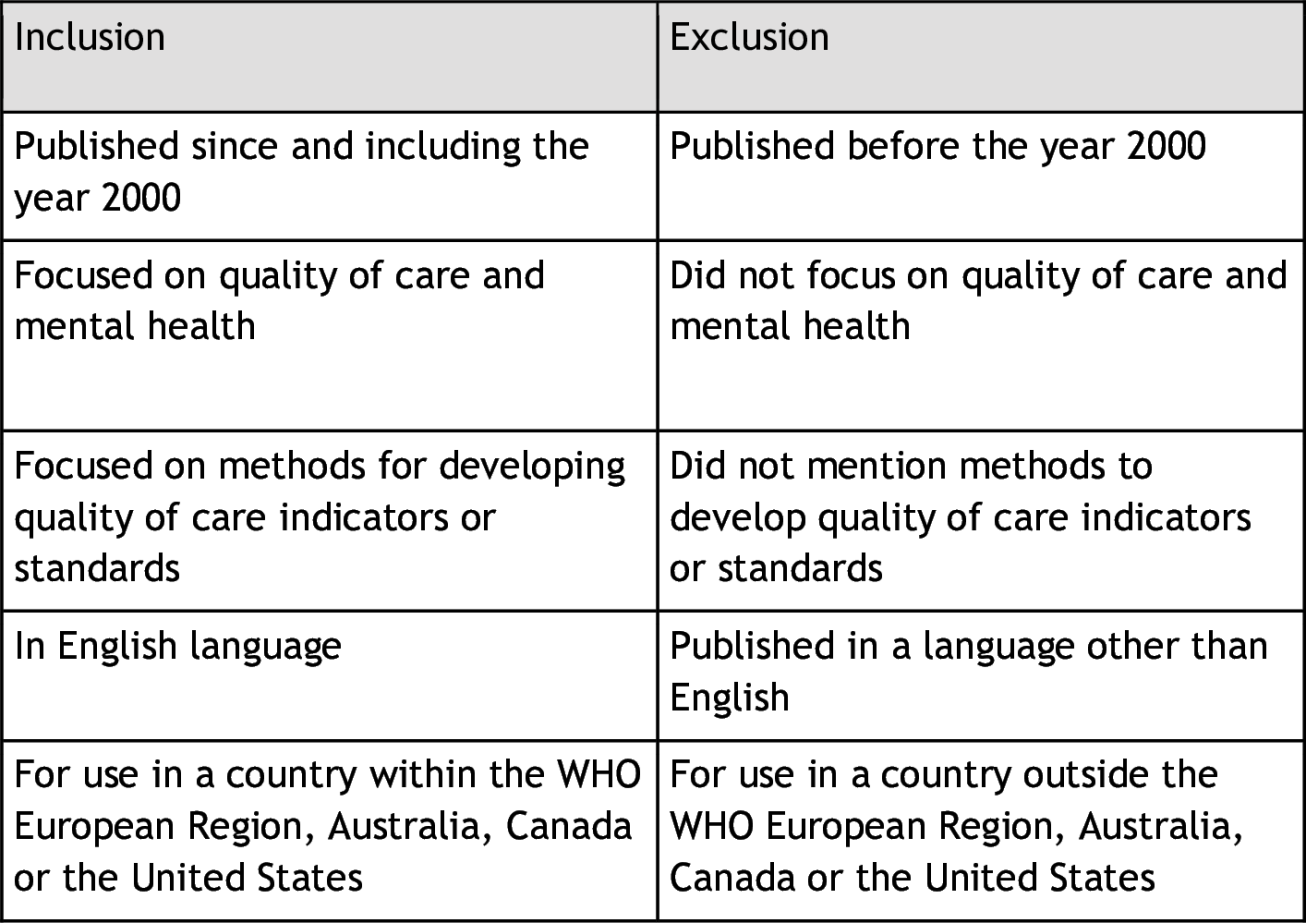
Inclusion/exclusion criteria.

The inclusion/exclusion criteria aimed to include papers that described the methods to develop quality indicators or standards for mental healthcare in the WHO European Region^21^, Australia, Canada or the United States. See figure 3 for inclusion/exclusion criteria.

### Data extraction and presentation

Data extraction was conducted by two independent researchers (JH and RS) using a data extraction tool created in Microsoft Excel. The headings in the data extraction tool were based on the proposed steps to develop quality measures for broader health by Dudley *et al*.^17^ Aligned with these steps, the following information was extracted: conceptual framework, methods to identify potential indicators/standards, criteria for selection of indicators/standards, expert or stakeholder consultations for selecting indicators, pilot or field test or implementation evaluation. Additionally, the following data were noted: title, first author, year of publication, country, aim of the study, indicators or standards, involvement of people with lived experience and rationale for overall methods used.

### Quality assessment

Study quality was evaluated by two researchers (JH and RS) using the *checklist for assessing the quality of qualitative studies.*^22^ Disagreements were resolved through discussion.

## Results

### Study selection

The literature search yielded a total of 678 publications: 207 from PubMed, 386 from Scopus, 13 from Psychinfo and 72 from Google Scholar. After removing 199 duplicate records, the initial pool was reduced to 479 titles and abstracts for screening; a further 393 articles were excluded as they did not meet the inclusion criteria. Full-text assessment was conducted for the remaining 86 studies: 66 studies did not meet the inclusion criteria and were excluded, while one was included from references. Ultimately, 21 studies^23^,^43^ were included in the rapid review (figure 2 for flow chart and online supplemental information for list of all studies).

### General characteristics of included articles

The articles included in the review were published between 2002^43^ and 2023^24^ with an even spread across the years. The papers aimed to develop quality standards/indicators for use in individual countries (n=17) or in multiple countries (n=4).^27 29 32 39^ Those that were for use in individual countries included those for use in the USA (23.8%, n=5^23 25 30 37 42^), Canada (n=4),^33^,^3541^ the UK (n=4),^31 36 40 43^ Italy (n=2),^24 26^ Norway (n=1)^28^ and the Netherlands (n=1).^38^ Research outlining the development of quality indicators/standards for mental healthcare was found in six out of the 56 countries in the inclusion criteria.

The quality assessment, conducted according to the *checklist for assessing the quality of qualitative studies,* revealed that all included studies were of high quality. Specifically, 16^2326^,^34 36^ studies achieved the maximum score of 1, four studies^24 35 39 41^ received a score of 0.9 and one study was rated as 0.8.^25^

Most of the included papers developed both quality standards and indicators (n=10),^2324 27 30^,^34 37 43^ followed by those that developed quality indicators alone (n=7)^26 28 29 35 38 40 41^ and those that developed quality standards alone (n=4).^25 36 39 42^

The included papers aimed to develop quality standards/indicators that assessed different aspects of mental healthcare. 11 aimed to assess the quality of specific mental health services: long-term residential units (n=3),^31 32 39^ specialised mental healthcare in hospitals (n=1),^34^ community mental health services (n=2),^25 37^ primary healthcare (n=2)^33 40^ and child and adolescent mental health services (n=3; one secondary care,^42^ one primary healthcare^36^ and one paediatric emergency unit, specifically looking at suicide-related behaviours^35^). Seven aimed to develop standards/indicators to assess the quality of care provided to populations with specific diagnoses (n=7): suicide prevention (n=2),^38 41^ bipolar (n=2),^24 30^ depression (n=1),^43^ schizophrenia (n=1)^26^ and co-occurring mental health and substance use conditions (n=1).^23^ Two papers aimed to develop quality standards/indicators to assess the national mental health system for international benchmarking^27 29^ and one paper aimed to assess the quality of referral letters between primary to secondary mental healthcare.^28^

The majority of papers (n=18) aimed to measure the quality of adult mental healthcare, with three focusing on the quality of child and adolescent mental healthcare.

All the general characteristics of the studies are presented in table 1.

**Table 1:** General characteristics of the included studies

Included studies (n=21)				
		N	%	
General characteristics	Country	5/21	23.8	The USA
		4/21	19.0	The UK
		4/21	19.0	Canada
		4/21	19.0	Multi
		2/21	9.5	Italy
		1/21	4.8	Norway
		1/21	4.8	Kingdom of Netherlands
	Types of services covered	10/21	47.6	Secondary and tertiary care
		5/21	23.8	All levels of care
		4/21	19.1	Primary care
		2/21	9.5	Tertiary care
	Quality measurement area	5/21	23.8	Quality assessment of a particular service
		5/21	23.8	International benchmarking of quality of care
		2/21	9.5	Quality of care received by a particular group of people (eg, marginalised populations)
		1/21	4.7	Quality of referrals
		8/21	38.1	Quality of care received by people with particular diagnosis (eg, treatment of depression, suicide prevention)

### Consistency of steps described

Most studies described either four or five of Dudley et al’s steps to developing quality indicators (71.4%) (see online supplemental material for list of all included papers and how they conform with the steps). Eight (38.1%) described all five steps,^23 26 31 34 36 37 42 43^ seven (33.3%) described four steps,^2829 33 35 38^,^40^ five (23.8%) described three steps,^25 27 30 32 41^ one (4.8%) outlined two steps^24^ and no papers described zero or one of the steps.

### Evaluation of methods used to develop quality measures for mental health

#### Rationale for the overall methods used

10 papers provided a rationale for the methods used: seven adapted previous methods,^2829 37^,^40 43^ one aimed to gather information from a range of different sources,^33^ one aimed to engage a wide range of stakeholders (n=1)^42^ and one aimed to prioritise inputs from caregivers (n=1).^36^

One paper^29^ described appointing a steering committee to guide the whole process, and one^42^ used participatory methods to involve stakeholders in the methods.

#### Conceptual framework (step 1)

All 21 studies outlined a conceptual framework; however, only three provided a conceptual framework with a named background or evidence.^28 29 32^

#### Methods to identify potential indicators/standards (step 2)

All studies described how potential indicators/standards were identified; however, there was great variation in how they were identified, and the level of detail in which this step was described.

The most common method to identify potential indicators/standards was through a literature review (n=11),^2425 28 31 34^,^36 38^ followed by a review of treatment guidelines (n=7),^2325^,^27 39 40 42^ a focus group or workshop (n=6),^28^,^3032 35 43^ review of existing quality standards, indicators and domains (n=4)^33 34 38 43^ and review of patient records (n=1).^41^ The vast majority of methods relied on pre-existing information only (n=14),^23^,^2733^ with fewer using methods that may uncover new concepts, such as focus groups or workshops (n=7).^28^,^3235 38^ Only seven of the articles involved people with lived experience of mental health services in the identification of potential indicators/standards.^2531 36 38^,^40 43^

#### Criteria for the selection of indicators/standards (step 3)

The criteria for the selection of indicators/standards were described by 15 (71.4%) of the studies.^2326 28 29 31 33^,^43^ There was variability in both the number and type of criteria used. Three used one criterion,^26 31 39^ five used two criteria,^35^,^3740 42^ three used three criteria^29 33 38^ and four used more than three criteria.^23 28 34 43^ The criteria used across the articles varied greatly, with a total of 21 different criteria being used (see table 2 for a full breakdown). The most commonly used criteria were *feasibility* (n=7),^23 26 28 29 34 38 42^ *importance* (n=7),^23 29 34 36 37 39 42^ *validity* (n=4),^23 28 33 40^ *relevance* (n=4)^33^,^3538^ and *appropriateness* (n=3).^31 37 43^

**Table 2:** Criteria used for selection of indicators/standard

Criteria used for selection of indicators/ standard	Number of studies that met step 3 and adopted the criteria
Feasibility	7
Importance	7
Validity	4
Relevance	4
Appropriateness	3
Scientific soundness	3
Measurability	2
Clarity	2
Reliability	1
Sensitivity to change	1
Acceptability	1
Simple	1
Communicable	1
Understandable	1
Variability	1
Action orientation	1
Usefulness	1
Practicality	1
Overall impression	1
Based on research evidence	1
Prioritised according to strength of research evidence and influence on outcome	1

#### Expert or stakeholder consultations for selecting indicators/standards (step 4)

Expert or stakeholder consultation to select indicators/standards was the third most described step (81.4%, n=17).^2325^,^29 31 32 34^ 10 studies specified what type of consensus method was used: Delphi was used by six,^3234 36 38^,^40^ RAND/UCLA by two^28 29^ and the Consensual Qualitative Research Method by one.^25^ Although most studies consulted with academic or health experts, nine consulted with people with lived experience of services (ie, children, young people, adults who have used services, caregivers) to select measures.^2531 32 36 38^,^40 42 43^

#### Pilot or field test or implementation evaluation (step 5)

Half (52.4%, n=11) of articles described a pilot, field test or implementation evaluation of the indicators/standards.^2324 30 31 33 34 36 37 41^,^43^ The studies used a variety of approaches, ranging from simpler methods, such as surveys sent via email, to more structured, elaborate ones involving complex statistical analyses. Two studies did not specify the type of pilot testing they conducted.^26 33^

#### Inputs from people with lived experience of mental health services

10 papers described the involvement of people with lived experience of mental health services in the development or prioritisation of standards/indicators.^2528 31 32 36 38^,^40 42 43^ None mentioned including people with lived experience in an overall steering group capacity, and one mentioned including people with lived experience to support with the design of methods as part of the participatory methods.^42^

## Discussion

The rapid review aimed to understand whether a systematic approach to developing mental health quality standards/indicators had been documented in published peer-reviewed and grey literature.

The included papers varied greatly in terms of theoretical underpinnings for conceptual frameworks, how potential quality measures were identified, the prioritisation of quality measures (who was consulted or how and what criteria were used) and how the quality measures were piloted, if at all.

These findings suggest that no consistent approach has been used to develop mental health quality standards/indicators. Hence, an easy-to-adapt pre-existing method to develop WHO quality standards for CAYMH services does not exist. This finding is consistent with other research, which found that no one unified approach was found to develop quality indicators for broader public healthcare.^17^

Less than half of the papers had any mention of the involvement of service users and/or caregivers in the process. This is in stark contrast to the growing consensus that users of health services should be involved in all decisions and processes to achieve high-quality care.^44 45^

Although 56 countries were represented in the inclusion criteria, the included papers were from high-income countries and predominantly from the UK, the USA and Canada. This finding is consistent with other research into the quality of mental healthcare which shows dominance from these countries.^15^

### Implications for policy and practice

Without a standardised approach to developing quality standards/indicators for mental healthcare, resulting quality indicators and standards across countries will likely differ in terms of whose definition of high-quality care is represented. To allow for cross-country and cross-service comparisons, there is a need for standardised methods to develop quality standards/indicators for mental healthcare. Policy makers, academics and people with lived experience should work together to develop methods that are pragmatic, feasible, evidence-based and allow for multiple voices to be represented, including those with lived experience.

The results of this review allow for some recommendations going forward to develop quality standards/indicators for mental healthcare. This includes the setup of an expert steering group^42^ to help guide the process and the development of a solid conceptual framework.^32^ A mixed methods approach to identifying potential standards/indicators for inclusion allows for a balance of learning from pre-existing standards/indicators, as well as allowing for new concepts to arise (eg, through focus groups^33^). Adapting previous methods to prioritise standards/ indicators (eg, the Delphi approach, adapted from RAND/UCLA),^2829 32 34 36 38^,^40^ allows for an evidence-informed approach to be taken. Consensus is needed in the criteria used to prioritise quality measures, with the most used ones in this review being feasibility and importance. The use of a pilot process provides information on barriers and enablers for implementation.^2324 30 31 33 34 36 37 41^,^43^

People with lived experience of health services should be involved in all activities that impact them.^44 45^ Hence, future methods should involve people with lived experience of mental health services at all stages, including in the development and oversight of methods (eg, steering group, participatory approach) as well as in the identification and prioritisation of potential quality standards/indicators and the pilot process.

This review has provided inspiration from which to develop methods for the WHO quality standards for CAYMH services. The first step will be to set up a steering group to oversee the overall methods. The overall approach will use an evidence-informed approach, which prioritises inputs and participation from a variety of stakeholders, including people with lived experience of mental health services. It is hoped that these learnings may provide inspiration for others looking to develop quality standards or indicators in mental healthcare and beyond.

There is a need for research on mental health quality standards/indicators from a wider variety of countries across the WHO European Region. Information sharing between countries can be used to spark the development and scale-up of such initiatives across the Region.

### Limitations

This study is subject to some limitations. First, we acknowledge the possibility of language bias, as only English-language studies were included in the review. Practical constraints, such as the difficulty of translating from various languages, influenced our decision to limit the study to English-language publications. Additionally, the exclusion of studies from regions outside the Region, Australia, Canada and the USA may limit the generalisability of findings to other countries and regions. Furthermore, by restricting the search strategy to electronic databases, there may be a risk of publication bias, as studies not published in peer-reviewed journals may have been overlooked.

## Conclusions

The review highlights the need to standardise the methods used to develop quality standards and indicators for mental healthcare and to prioritise inputs from people with lived experience. Recommendations going forward to develop quality standards and indicators have been developed, as well as the need for more research in this area.

## Supplementary material

10.1136/bmjoq-2025-003533 online supplemental file 1

## Data Availability

All data relevant to the study are included in the article or uploaded as supplementary information.
