# Supplementary material for: Methods used to develop quality of care standards and indicators for mental health across the WHO European region: a rapid systematic review
Source: BMJ Open Qual. 2025 Dec 9;14(4):e003533. doi: 10.1136/bmjoq-2025-003533 (PMC12699667; doi:10.1136/bmjoq-2025-003533)
Supplement: online supplemental file 1 [file bmjoq-14-4-s001.docx]

Supplementary information: All included papers against Dudley et. al’s criteria

| Title | Authors | Language | Year | Country | Aim of the Study | Rationale for methods | Indicators or Standards | Step 1_Conceptual framework | Step 2_Methods to identify potential indicators/ standards | Step 3_Criteria for selection of indicators/ standards | Step 4_Expert or stakeholder consultations for selecting indicators | Step 5_Pilot or field test or implementation evaluation |
| --- | --- | --- | --- | --- | --- | --- | --- | --- | --- | --- | --- | --- |
| Quality indicators for the referral process from primary to specialised mental health care: an explorative study in accordance with the RAND appropriateness method | Hartveit M. et al. | English | 2017 | Norway | To develop quality indicators to detect the impact that the quality of referral letters from primary care to specialised mental health care has on the quality of mental health services. | Adaptation of RAND / UCLA appropraiteness method | Sixteen preliminary indicators emerged during the focus group interviews and literature review; the expert panels recommended four of the 16 indicators. | Yes – relevance of quality of referral for overall quality of care. | Yes (Systematic literature review and foucus group; the focus group were composed by health professionals, patient representatives and managers). | Yes Validity, Reliability, Sensitivity to change, Acceptability, Feasibility, Simple and Communicable | Yes (RAND/UCLA).  No involvement of patients/carers | No |
| Individual, programmatic and systemic indicators of the quality of mental health care using a large health administrative database: an avenue for preventing suicide mortality | Thibodeau L. et al. | English | 2018 | Canada | To describe the development of quality of care indicators -that may improve strategies for suicide prevention- at the three levels (individual, program, and system) of the quality of mental health care services model and the corresponding statistical analysis strategies designed. | No | Thirteen proposed candidate quality indicators at the programmatic and system levels, supported by the health services and public health literature or practices. | Yes, clearly labelled for the “untapped potenital of linked health administrative databases for identifying suicide prevention avenues”. Adapted previous model (Tansella and Thornicroft). | Yes using large linked health administrative database from the province of Quebec (QICDSS); literature review of health services research; practices from epidemiological studies. | No | No  No involvement of patients/carers | Yes  (Several statistical models will be tested to explore the relationship between changes in suicide rates and each individual, program and system level indicators of the 13 candidate indicators). |
| The mental health and addictions quality initiative: collaboration in public reporting and quality improvement | Prince P. et al. | English | 2014 | Canada | Standardized mental health and addictions indicators reflective of hospital accountability and accessible to the public were developed and are currently compared among 15 hospitals. | No | 15 quality indicators to measure 9 quality domains. | Yes | Yes (Informal survey of peer reviewed and grey literature describing general and mental health facility domains and indicators(e.g. local, national and international reports, strategic plans). | Yes Selection of criteria for assessing potential indicators: a. relevance and importance; b. scientific soundness; c. feasibility; d. understandable and meaningful from a public perspective. Hospitals. | Yes face to face-to-face Delphi completed by hospital boards at different facilities, including administrative and clinical leaders. No involvement of patients/carers. | Yes  (The hospitals worked together to populate draft scorecards with those indicators that were initially deemed feasible while pursuing improved data quality for RAI-MH indicators. Indeed, comparing and contrasting facility results at face-to-face meetings between Chief Executive Officers (CEOs) and clinical, decision support, technical, and other experts facilitated improvement in the reliability of data collected). |
| Quality indicators for international benchmarking of mental health care | Hermann R. et al. | English | 2006 | Multi | The Organization for Economic and Community Development’s Health Care Quality Indicators Project (OECD–HCQI) is the first effort of which we are aware to identify measures for international benchmarking of quality of mental health care. This article reports on the methods employed to develop consensus among participants along with the resulting measures, as well as challenges to be surmounted for further progress to be achieved. | Yes – the use of Dephi method becuase it has previously been applied to the selectino of quality measrues. | Twelve indicators / measures proposed. | Yes, outlined rationale. | Yes  (indicators were drawn from OECD member countries’ initiatives;Canadian Mental Health Advisory Network, the United Kingdom Department of Health, the Center for Quality Assessment and Improvement in Mental Health’s National Inventory of Mental Health Quality Measures, numerous US stakeholder initiatives, and published research reports). To be included measures had to focus on quality, be relevant to assessing quality at the system level, focus on technical quality, be constructed from pre-existing administrative data based on standardized coding, and be a single-item indicator. No involvement of patients/carers | Yes  Conducted over two phases – panelists anonymously rated measures for importance, scientific soundness and feasibility; for measures were consensus was lacking, a second review was conducted making decisions about inclusion/exclusion on a case by case basis.  Panel members were those nominated from UK, Sweden, Canada, Australia, Denmark, the United States and the European Society for Quality in healthcare. They included mental health administrators, clinicians, researchers.  No service users. | Yes – adapted RAND/UCLA appopriatneess method where scores of 7-9 indicated agreement, 4-6 neither agreement or disagreement, and 1-3 disagreement. Measures with a median socre of 7 or more for importance and soundness were included, and for which more than half of panelists reported data avilability was ‘possible’ or ‘likely’.  No involvement of patients/carers | No |
| Quality assessment of primary care for common mental disorders in isolated communities: Taking advantage of health records | [Lessard](https://pubmed.ncbi.nlm.nih.gov/?term=Lessard+L&cauthor_id=26164064) L. et al. | English | 2015 | Canada | The goal of this study was to identify useful, measurable and valid indicators to assess the quality of primary health care offered to people with depression or anxiety in remote regions, based on information found in health records | Yes – wanted to use a hybrid apporach to integrate evidnece, opinions from key informers and case studies to result in an assessment citeira that would be both suitable for remote isolated regions and allow comparison. | Eighteen quality indicators proposed to measure four recommended dimensions of primary health care clinical processes: recognition, assessment, treattment and follow-up. | Yes | Yes (Guidelines for depression and anxiety disorders and indicators from the National Inventory of Mental Health Quality Measures (NINHQM); institutional records; case studies). | Yes  The adaptation to rural isolated communities was appraised according to their relevance, measurability and construct validity in this context, by three members of the mental health team from two participating health centres in face to face to meetings. | No  No involvement of patients/carers | No |
| Quality of care indicators for schizophrenia: determinants of observed variations among Italian Departments of Mental Health. Results from the ETAS DSM study | [Fantini](https://pubmed.ncbi.nlm.nih.gov/?term=Fantini+G&cauthor_id=27019391) G. et al. | English | 2017 | Italy | The primary aim of this study is to analyse the conformance of usual care patterns for persons with schizophrenia to treatment guidelines in three Italian Departments of Mental Health (DMHs). The secondary aim is to examine possible organisational and structural reasons accounting for variations among DMHs. | No | Nine pharmacological indicators and nine non pharmacological indicators proposed. | Yes, need to benchmark the perofrmance of mental health services in different regions to identify discrepancies, health outcomes, and regional use of resources. | Yes (Literature and guidelines) No involvement of patients/carers | Yes - (Feasibility) | Yes indicators selected by 30 health care professionals of participating institutions in person by marking the feasibility of implementation.  No involvement of patients/carers | Yes outlines the implementation of the quality of care indicators and the success of it. |
| Perspectives on quality mental health care from Brazilian and Cape Verdean outpatients: implications for effective patient-centered policies and models of care | De Jesus M. et al. | English | 2014 | USA | The aim of this study was to define indicators of “good” quality care from the perspective of Brazilian and Cape Verdean immigrant patients. | No | Three primary domains were proposed with nine sub categories. | Yes, the study fills a gap in the literature to examine perceptions of quality of care in two underrepresented patient groups, to support the development of more culturally appropratie and effective policies and practices. | Yes Involvement of patients focus group | No | Yes (Consensual Qualitative Research method) Involvement of patients in reviewing the result | No |
| The development of the Quality Indicator for Rehabilitative Care (QuIRC): a measure of best practice for facilities for people with longer term mental health problems | [Killaspy](https://pubmed.ncbi.nlm.nih.gov/?term=Killaspy+H&cauthor_id=21362167) H. et al. | English | 2011 | Multi | To develop an international, standardised toolkit to assess the quality of care in longer term hospital and community based mental health units, including the degree to which human rights, social inclusion and autonomy are promoted. | No | Nine domains included in the toolkit, 154 questions. | Yes | Yes  (Literature review, international expert Delphi exercise and a review of care standards in ten European countries)  No involvement of patients/carers | No | Yes the project steering committee reviewed findings and agreed on domains for inclusion in the toolkit.  No service users involved. | Yes – inter rater reliability testing of the draft toolkit was tested in 202 facilities. The toolkit was updated based on feedback from interviewers and unit managers, inter-rater reliability testing and results of the exploratory factor analysis. Final version comprised 145 questions assessing seven domains of care. |
| The quality of mental health care for people with bipolar disorders in the Italian mental health system: the QUADIM project | [D'Avanzo](https://pubmed.ncbi.nlm.nih.gov/?term=D%27Avanzo+B&cauthor_id=37312076) B. et al. | English | 2023 | Italy | To evaluate the quality of mental healthcare delivered to patients with bipolar disorders taken-in-care by Italian public services of mental health, using healthcare utilization databases. | No | Thirty-six quality indicators were developed and grouped in three categories of Accessibility and Appropriateness (n = 28), Continuity (n = 4) and Safety (n = 4) describing aspects of mental health care for people with bipolar disorders. | Yes | Yes (Recommendation of the agreement between the Ministry of Health and the Italian Regional Governments, considering the guidelines developed by the Canadian Psychiatric Association and the National Institute for Clinical Excellence). | No | No  No involvement of patients/carers | Yes- implementation was evaluated. |
| The use of a participatory approach to develop a framework for assessing the quality of care in children's mental health services | [Vargo](https://pubmed.ncbi.nlm.nih.gov/?term=Vargo+AC&cauthor_id=22527710) A. et al. | English | 2013 | USA | The purpose of this study was to develop a framework for assessing the quality of children’s mental health services that reflects the primary concerns and perspectives of diverse stakeholders. | Yes – wanted to engage stakeholder in identifying measurement indicators and data sources. | Final Quality of Care Framework contained twenty three sub components split across four main domains (access, appropriateness, consumer engagement and outcomes) across two main areas (service quality and outcomes). | Yes, fills a gap in that most mental health quality measures are geared towards adults and without invovlement of stakeholders. | Yes  Interviewed poviders and caregivers, literature review and of national standards, initial indicator rating.  (Literature) | Yes  (Importance, Feasibility) | Yes, stakeholder meetings with participation from administarotrs and staff to solicit feedback on on feasibility of indicators.  Involved caregivers of r children with mental health conditions. | Yes  (Mail survey instrument for assessing quality of care based on the developed framework) |
| Developing measures of quality for the emergency department management of pediatric suicide-related behaviors | [Rhodes](https://pubmed.ncbi.nlm.nih.gov/?term=Rhodes+AE&cauthor_id=23114232) A. et al. | English | 2012 | Canada | To identify key candidate process indicators (quality of care measures) and structural measures (organizational resources and attributes) important for emergency department (ED) management of pediatric suicide-related behaviors. | No. | Of the 42 candidate process indicators, those identified as both most relevant to patient care (Q87% agreed or strongly agreed) and most variable across hospitals (Q78% agreed or strongly agreed) were selected (n=9). Four indicators from hospital and regional resources were identified as opportunities for improvement. | Yes, suicide-related behaviours are an important public health issue and there is a lack of quality of care measrues. | Yes (Guidelines, published studies). No involvement of patients/carers | Yes (Relevance and Variability). | Yes (Dillman method via survey of expert pediatric ED clinicians). No involvement of patients/carers | No |
| Quality indicators for primary care mental health services | [Shield](https://pubmed.ncbi.nlm.nih.gov/?term=Shield+T&cauthor_id=12679505) T. et al. | English | 2003 | UK | To identify a generic set of face valid quality indicators for primary care mental health services which reflect a multi-stakeholder perspective and can be used for facilitating quality improvement. | Yes, two round postal Delphi survey. | The indicators have been categorised into 21 aspects of care, 11 relating to general practice and 10 relating to health authorities or primary care groups/trusts. | Yes | Yes (guidelines statements and quality indicators and standards relating to primary mental health care; published/grey literature). Involvement of patient focus group | Yes (Validity and Clarity) | Yes (Delphi). Involvement of carers and patients | No |
| Prioritizing suicide prevention guideline recommendations in specialist mental healthcare: A Delphi study | [Setkowski K](https://www.scopus.com/authid/detail.uri?authorId=57191037322). et al. | English | 2020 | Netherlands | This study aimed to prioritize suicide prevention guideline recommendations and to develop a set of quality indicators (QIs) for suicide prevention in specialist mental healthcare. | Yes, delphi technique has been used to select quality indicators in the past and is a structured, iterative process to reach consensus. | Final list of relevant, actionable and feasible quality indicators derived from the guideline consisted of eleven indicators. | Yes | Yes (guideline recommendations for the diagnosis and treatment of suicidal behaviour, healthcare professionals discussed the lists resulting in 11 quality indicators). | Yes (Relevance, Action orientation and Feasibility) | Yes (elecontric survey to do the delphi method, involvement of suicide experts, patient advisory boards, experts with experiences in suicidal behaviour, healthcare professionals).If 70% of responses were within the range of median scores of 4 and 5, were included. Involvement of patients | No |
| Developing mental health-care quality indicators: Toward a common framework | Fisher C. et al. | English | 2013 | Multi | This report describes an initial component of a project undertaken by an international collaborative group that aims to develop a common framework of measures that will allow for international comparisons of mental health system performance. | No | Indicators from 31 programs in 11 countries and two cross-national programs were compiled, yielding 656 total measures. The final framework comprised 17 domains and 80 subdomains. Each indicator specified an objective numerator and denominator drawn from an identifiable dataset. | Yes | Yes  (Peer-reviewed journal articles, government reports, white papers and other ‘gray literature’ on population-based quality or performance measurement initiatives in mental health being developed or implemented in each country at the national or other representative level). No involvement of patients/carers | No | Yes (method not specified)  No involvement of patients/carers | No |
| Quality standards for child and adolescent mental health in primary care | Sayal K. et al. | English | 2012 | UK | This study uses a parent/caregiver led approach to develop a measure for assessing and improving the quality of care for children with mental health problems. Based on parental views, it aimed to develop parent/caregiver derived quality standards for primary care child and adolescent mental health and to develop consensus about the importance of these quality standards within primary care. | Yes, used iteartive approach and delphi methods, prioritising inputs from parents. | Ten quality standards resulted across healthcare domains involving access, confidentiality, practitioner knoweldge, communication, continuity of care and referral to other services. | Yes | Yes Involvement of parents, representatives from the voluntary sector, children and young people's services and Child and Adolescent Mental Health Services | Yes  (Clarity and Importance durig the first 2 phases of the process; only Importance has been used again during the following Delphi phase) | Yes (Delphi) Involvement of parents and representatives from voluntary organisations | Yes (  The final set of Quality Standards was piloted with parents at two GP practices to assess their feasibility in practice and to ascertain test-retest reliability) |
| Implementing composite quality metrics for bipolar disorder: Towards a more comprehensive approach to quality measurement | [Kilbourne A.](https://www.scopus.com/authid/detail.uri?authorId=7004062071) et al. | English | 2010 | USA | Implementing a set of processes of care measures for bipolar disorder that reflect psychosocial, patient preference, and continuum of care approaches to mental health, and examining whether veterans with bipolar disorder receive care concordant with these practices. | No | Indicators were categorized into four categories and composite measures of quality were developed based on the above-mentioned four categories. | Yes, the need for process quality measures for quality of care for bipolar disorder. | Yes (Treatment guidelines for bipolar disorder developed by the American Psychiatric Association, the Standards for Bipolar Excellence (STABLE) project, and the RAND-Altarum national evaluation of VHA mental health programs). No involvement of patients/carers | No | No  No involvement of patients/carers. | Yes  (Baseline survey of veterans hospitalized/attending their outpatient MH appointment at a large VA MH facility) |
| Performance measurement for co-occurring mental health and substance use disorders | Dausey D. et al. | English | 2009 | USA | The goal was to develop a small number of measures that could serve as examples of the types of measures for co-occurring mental health and substance use disroders that could be pilot tested and refined for future use. | No | A total of 36 performance measures, broken into structures, processes, and outcomes -with at least one measure for each subcategory identified- were developed. | Yes | Yes (existing evidence-based measures) No involvement of patients/carers | Yes (Importance, Usefulness, Validity or Scientific Soundness, Practicality or Feasibility, Overall) by expert panel including indiviudals with leardership roles at state level, individuals with roel in perofrmance measurement and academic persectives. None with lived experience. | Yes (method not specified) No involvement of patients/carers. | Yes  (Twelve measures that appeared the most promise to pilot test were chosen) |
| The development of a set of quality indicators to evaluate services for people with depression | [Worral A](https://www.scopus.com/authid/detail.uri?authorId=7004022722). et al. | English | 2002 | UK | In this article the authors describe the process of developing a set of quality indicators to support and guide the CSAG evaluation of services. They had two aims in developing the quality indicators: firstly, to generate a broad range of quality indicators to encompass all aspects of service provision relevant to the care of people with depression; and secondly, to contribute towards the development of a definitive set of service standards that could be used in local primary care focused service evaluations. | Yes- adapted RAND/UCLA Delphi approach. | The 104 final quality indicators was categorised within four themes or ‘levels’ -describing different ‘actors’responsible for the action covered by the indicator, and ‘customers’ who are the immediate beneficiaries- relating to the treatment and management of depression | Yes | Yes (Literature review; workshop of different stakeholders -including users and carers) | Yes Baker&Fraser (1995) attributes of criteria for assessment of quality: - Based on research evidence - Prioritised according to strength of research evidence and influence on outcome - Measurable-clear and precise - Appropriate to the clinical setting. | Yes (method not specified) Consensus within each mixed group, including users and carers. | Yes  (A 1-day visit to a mental health trust was arranged to pilot the quality indicators. This piloting and the subsequent editing resulted in a final set of 104 quality indicators, which were used in the CSAG depression study) |
| Adaptation of the Quality Indicator for Rehabilitative Care (QuIRC) for use in mental health supported accommodation services (QuIRC-SA) | Killaspy H. et al. | English | 2016 | UK | This paper reports on the first work package (WP1), the adaptation of an existing quality assessment tool (the Quality Indicator for Rehabilitative Care, QuIRC) for mental health supported accommodation services. | No | A total of 28 QuIRC items were rephrased, 20 items were deleted and 10 items were added. The final version had 143 full items across seven domains. | Yes | Yes (QuIRC)  No involvement of patients/carers. | Yes (Appropriateness) | Yes (method not specified)  Involvement of members with lived experience of specialist mental health supported accommodation and services and members with with lived experience of mental health problems and expertise in mental health services research. | Yes  (The adapted QuIRC was piloted with three service managers) |
| Developing quality indicators for family support services in community team-based mental health care | Olin S. et al. | English | 2013 | USA | This paper provides a description of the variation in performance on the QI sets and explores the utility of these indicators for measuring quality of family support services within children’s mental health. | Yes a modified Delphi approach. | This process resulted in 14 Program Quality Indicators and 27 quality domains. | Yes | Yes (Documents and guidelines for activities) | Yes (Importance, Appropriateness) | Yes (method not specified) No involvement of patients/carers. | Yes  (The utility of these QIs were assessed by applying them to data previously collected from 21 HCBS Programs) |
| Promoting Recovery in Long-Term Institutional Mental Health Care: An International Delphi Study | [Turton](https://pubmed.ncbi.nlm.nih.gov/?term=Turton+P&cauthor_id=20194407) P et al. | English | 2010 | Multi | This study aimed to identify specific items of care that key stakeholders regard as most important in promoting recovery for people with longer-term mental health problems in inconstitutional care. | Yes, delphi. | Items achieving high median ratings plus high consensus ratings—many of which were similar across groups—were organized into 11 broad domains of care. | Yes | Yes  Focus group including users, caregivers and advocates. | Yes (Importance) | Yes (Delphi) Involvement of service users, caregivers, and advocates | No |
